# Comprehensive transcriptome and metabolome analysis to exploration the effects of TCs on GCs lipid metabolism at goose pre-ovulatory follicle

**DOI:** 10.1371/journal.pone.0340283

**Published:** 2026-01-09

**Authors:** Jisi Ma, Wenqiang Sun, Xin Yuan, Hongyu Long, Hui Shen, Chunying Liu, Xiang Gan

**Affiliations:** 1 College of Basic Medicine, Guilin Medical University, Guilin, Guangxi, China; 2 College of Animal Science and Technology, Sichuan Agricultural University, Chengdu, Sichuan, China; 3 Scientific Research Center, Guilin Medical University, Guilin, Guangxi, China; 4 College of Nursing, Guilin Medical University, Guilin, Guangxi, China; King Saud University / Zagazig University, EGYPT

## Abstract

Granulosa cells (GCs) and theca cells (TCs) have complex communication and interactions. Lipid metabolism in GCs is important for follicle development, which is also inseparable from the role of TCs; however, the underlying mechanisms remain unclear. In this study, we selected the pre-ovulatory (F1) follicle of geese—a poultry species with relatively low egg production—which exhibits high lipid content and vigorous lipid metabolism. Using transcriptomic and metabolomic approaches, we analyzed the effects of TCs on GCs in a co-culture model. We identified and screened the core functions and signaling pathways associated with the differentially expressed metabolites (DEMs) and genes (DEGs) between the mono-culture and co-culture groups. Key metabolites and genes within the core pathway were subsequently validated using ELISA and qPCR. Both transcriptomic and metabolomic results showed that after co-culture, the metabolite and gene expression profiles of GCs were significantly altered. Enrichment analysis revealed that both DEMs and DEGs were significantly associated with sphingolipid and glycerophospholipid metabolism pathways. In the co-culture group, metabolomic and ELISA results indicated that the concentrations of core metabolites, phosphorylcholine (PC) and phosphatidylethanolamine (PE), in GCs increased significantly (*p* < 0.05). Transcriptomic and qPCR results both showed that the expression levels of genes involved in PC catabolism, including phospholipase A2 epsilon-like-1, cytosolic phospholipase A2 epsilon-like-2, and phospholipase A2-like genes in GCs decreased significantly (*p* < 0.05). In summary, our results suggest that TCs may affect the lipid metabolism of GCs in goose F1 follicles by promoting PC synthesis within the glycerophospholipid metabolism pathway.

## Introduction

The granulosa cell (GC), as a component of the follicle, plays an important role in the recruitment, growth, selection, and maturation of follicles by providing support, nutrition, and secretory factors [1,2]. In mammals, numerous studies have shown that the proliferation, apoptosis, and steroid synthesis of GCs play an important role in follicular development [3–5]. Recently, a growing number of studies have shown that lipid metabolism in GCs also has important implications for follicular development [3–6]. In cattle, sheep, and humans, lipid metabolism of GCs also plays an important role in follicular development and oocyte maturation [7–11], particularly in transporting nutrients and exchanging signals ^[^12^]^. Unlike in mammals, poultry follicles contain yolk, which is rich in lipids. Therefore, lipid metabolism in GCs is likely to be even more critical and intriguing during follicular development in birds. However, lipid metabolism in avian follicles has rarely been reported. Our published research demonstrates that de novo lipogenesis (DNL) exists in poultry GCs and may play an important role in their lipid metabolism [13]. It is evident that lipid metabolism in GCs is more complex during the development of poultry follicles than in mammals.

The theca cell (TC) is also a component of follicles. TCs and GCs are located on opposite sides of the follicular basal lamina and therefore communicate across it primarily via endocrine mechanisms [14–16]. The influence of TCs on GCs persists throughout follicular development. Through paracrine signaling, TCs maintain dynamic communication with GCs, supporting the normal development of both GCs and the follicle [17–19]. Similarly, lipid metabolism in GCs is also regulated by TCs. However, in avian species, TCs have a unique regulatory pattern for GCs, which is significantly different from that in mammals. This uniqueness further limits our exploration of lipid metabolism patterns in poultry GCs. In a previous study, we simulated in vivo conditions by establishing an in vitro co-culture model of GCs and TCs. We found that TCs had a significant impact on the physiological properties of GCs at all stages of follicular development, including their DNL process [20]. Moreover, in pre-ovulatory (F1) follicles, TCs can act on GCs and lead to significant downregulation of *FAS* and *ACC*, which are key genes in the DNL synthesis pathway in GCs [20]. Obviously, TCs play a significant role in lipid metabolism in GCs. However, previous studies have been limited to the DNL process. The broader impact of TCs on lipid metabolism pathways in GCs and the underlying regulatory mechanisms remain unclear.

In this study, we focused on the F1 follicles, which exhibit the highest lipid content, deposition, and metabolic activity. Employing our established GCs-TCs co-culture model, we combined transcriptomic and metabolomic analyses to identify the lipid-related molecules and pathways in GCs that are influenced by TCs. This approach allowed us to elucidate the pattern by which TCs regulate lipid metabolism in GCs within goose F1 follicles. The findings of this work are expected to provide a theoretical foundation for understanding the mechanisms of follicular development in poultry.

## Materials and methods

### Experimental animals

This study utilized healthy laying Tianfu meat geese (Anser cygnoides) from a maternal line, aged 35 to 45 weeks. The geese were housed under natural light and temperature conditions at the Experimental Farm for Waterfowl Breeding of Sichuan Agricultural University (Sichuan, China) and had free access to feed and water. The laying cycle of each goose was recorded. Geese in the same laying cycle were euthanized by cervical dislocation 7–9 h before the expected oviposition time. All animal procedures were approved by the Laboratory Animal Welfare and Ethics Committee of Sichuan Agricultural University (Permit No. 20220154).

### Separation of goose follicle GCs and TCs at F1 stage

F1 follicle [21] were dissected from the ovaries and rinsed with ice-cold sterile phosphate-buffered saline (PBS, pH 7.4, Solarbio). Connective tissue was carefully peeled away with tweezers, and a slit (approximately 0.5–2.0 cm long) was made on the side opposite the stalk using a surgical blade, allowing the yolk and the granulosa layer to flow out. The granulosa and theca tissues were then separated and washed repeatedly with PBS to remove residual yolk. Subsequent isolation and culture of GCs and TCs were carried out following our established protocols [21,22].

### Mono-culture and co-culture of goose GCs

Granulosa cells (GCs) from F1 follicles were seeded in six-well plates (Corning) at a density of 1.2 × 10⁶ cells per well in 2.5 mL of DMEM/F-12 medium (HyClone). Theca cells (TCs) from the same follicle stage were seeded onto transwell inserts (pore size: 0.4 μm; Corning) placed in the same six-well plates at 1 × 10⁶ cells per insert in 1.5 mL of medium. All cells were cultured at 37°C under 5% CO₂ in a humidified atmosphere. The medium was replaced after 6–8 hours, once cell adhesion was confirmed. For the co-culture group, GCs and TCs from the same follicle were cultured together in the same plate system, with the start of co-culture designated as 0 hours. In the mono-culture control group, GCs were cultured with a cell-free transwell insert containing 1.5 mL of medium [23].

### Transcriptome analysis

Total RNA was extracted from the cells. RNA concentration and integrity were assessed using an Agilent Bioanalyzer 2100 system (Agilent Technologies, CA, USA). Then, 1–4 μg of RNA per sample was used as input material for transcriptome library construction. Following quality control, qualified libraries were pooled based on their effective concentration and the desired sequencing depth. The pool was then sequenced on an Illumina platform to generate 150 bp paired-end reads. Clean reads were obtained by filtering out adapter-containing reads, poly-N reads, and low-quality reads from the raw data. The reference genome index was built using HISAT2 (v2.0.5), and the clean reads were aligned to the goose reference genome. The resulting mapped reads for each sample were assembled using StringTie (v1.3.3b) in a reference-guided manner [24]. Finally, the FPKM (Fragments Per Kilobase of transcript per Million mapped reads) value for each gene was calculated based on the gene length and the mapped read count. Differentially expressed genes (DEGs) were identified using the DESeq2 R package (v1.20.0). Genes with an absolute log2 fold change ≥ 1 and an adjusted p-value ≤ 0.05 were considered differentially expressed. Gene Ontology (GO) and Kyoto Encyclopedia of Genes and Genomes (KEGG) pathway enrichment analyses of the DEGs were performed using the clusterProfiler R package.

### Metabolomics analysis

A 100 μL aliquot of each sample was mixed with 400 μL of extraction solution (MeOH:ACN, 1:1, v/v), which contained deuterated internal standards. The mixture was vortexed for 30 s, sonicated for 10 min in an ice-water bath, and then incubated for 1 h at −40°C to precipitate proteins. The samples were then centrifuged at 12,000 rpm (RCF = 13,800 × g, r = 8.6 cm) for 15 min at 4°C. The supernatant was transferred to a fresh glass vial for analysis. The quality control (QC) sample was prepared by combining equal aliquots of the supernatant from all individual samples.

For polar metabolites, LC-MS/MS analyses were performed using a UHPLC system (Vanquish, Thermo Fisher Scientific) equipped with a Waters ACQUITY UPLC BEH Amide column (2.1 mm × 50 mm, 1.7 μm) coupled to an Orbitrap Exploris 120 mass spectrometer (Thermo Fisher Scientific). The mobile phase consisted of (A) 25 mmol/L ammonium acetate and 25 mmol/L ammonium hydroxide in water (pH = 9.75) and (B) acetonitrile. The auto-sampler temperature was maintained at 4 °C, and the injection volume was 2 μL. MS/MS spectra were acquired using an information-dependent acquisition (IDA) mode controlled by the Xcalibur software (Thermo), which continuously evaluates the full scan MS spectrum. The electrospray ionization (ESI) source conditions were set as follows: sheath gas flow rate, 50 arb; auxiliary gas flow rate, 15 arb; capillary temperature, 320 °C; full MS resolution, 60,000; MS/MS resolution, 15,000; collision energy, stepped NCE 20/30/40; and spray voltage, 3.8 kV (positive) or −3.4 kV (negative).

The raw data were converted to mzXML format using ProteoWizard, and then XCMS software was used for peak alignment, retention time correction, and peak area extraction. The data extracted by XCMS were first used for metabolite structure identification and data preprocessing, then for experimental data quality evaluation, and finally for data analysis. Local self-constructed databases and public repositories, including the Human Metabolome Database (HMDB) (http://www.hmdb.ca), Metlin (http://metlin.scripps.edu), MassBank (http://www.massbank.jp/), and mzCloud (https://www.mzcloud.org), were used for database searching. Metabolites in the biological samples were structurally identified by comparing their retention times, molecular masses (with a mass error of <10 ppm), secondary fragmentation spectra, collision energy, and other relevant information against the databases. The identification results were then analyzed. Metabolites identified at Level 2 or above were subjected to orthogonal partial least squares discriminant analysis (OPLS-DA) to identify potential biomarker variables. Significantly different metabolites between groups were determined based on a variable importance in projection (VIP) ≥ 1 and an adjusted P-value ≤ 0.05. Hierarchical clustering analysis was performed using R (http://www.r-project.org/).

### Isolation of total RNA and quantitative real-time PCR

Trizol (Invitrogen) was used to isolate total RNA from GCs cultured to the F1 stage at 48 h. The RNA was reverse-transcribed into cDNA using a PrimeScript™ RT reagent kit (TaKaRa, Japan) according to the manufacturer’s instructions. Quantitative real-time PCR (qPCR) was performed on a CFX96™ Real-Time system (Bio-Rad, USA) with SYBR® Premix Ex Taq™ (TaKaRa). Each 25 μL qPCR reaction contained 2.0 μL of cDNA, 12.5 μL of SYBR Premix Ex Taq, 1.0 μL each of forward and reverse primers, and 8.5 μL of sterile distilled water. All reactions were performed in three technical replicates [23]. The expression levels of the target genes were normalized to glyceraldehyde-3-phosphate dehydrogenase (GAPDH) and calculated using the 2^–△△Ct^ method [25]. The sequences of the primers used are listed in Table 1.

**Table 1:** Primer pairs for real-time quantitative PCR.

Primer name	Primer sequence (5’– 3’)	Product size (bp)	Tm (ºC)
Cytosolic phospholipase A2 epsilon-like-1-F	TGAGCCAGGAGGACAGAGAACA	129	60
Cytosolic phospholipase A2 epsilon-like-1-R	GCTCCACATCTGGTGCCTTGT		
Cytosolic phospholipase A2 epsilon-like-2-F	CAAGACCACGAGGTGCCGATAG	143	60
Cytosolic phospholipase A2 epsilon-like-2-R	GTGCCAGATGAGCCACTGATGT		
DGKI-F	CCAGATCGCCTGCGGATTAGAG	145	60
DGKI-R	TCCGACAGGTCTCCAAGTCACA		
DGKQ-F	TGGATCAGCGAGAGGTGGAGTT	109	60
DGKQ-R	AAGCGGTCATCACTGTCAGAGC		
Ethanolamine phosphotransferase 1-like-F	TGGTGTGGAAGTTTGGCAGAAG	197	60
Ethanolamine phosphotransferase 1-like-R	GCAACAGAGGTGACACAAGAGG		
Phospholipase A2-like-F	ACGACTGCTGCTACGACAAGG	192	60
Phospholipase A2-like-R	AGAGGGTGAAGAGCGGGTTGTA		
ASAH2-F	CCACCACCAACCCAGAAACCAT	159	60
ASAH2-R	TCGGCTGTAGAGGCGTGTGA		
NEU2-F	TGGCTTCGTTTCCTGTCCTGAA	142	60
NEU2-R	TGTTCGTCCACCACATCCTCTC		
SPTLC3-F	GTGCGAGGCTTTCAGGTGCTA	120	60
SPTLC3-R	TTCTCCATGCTCTGCGGCTTC		
STAR-F	AGAATCTTGACCTCTTTGACGCTG	87	60
STAR-R	GAGACGGTGGTGGATAACGGA		
ABCA5-F	GACGGCATCATCACGGAGGAAT	112	60
ABCA5-R	GCTGGTAGGACATGGCATCGTT		
HSD11B2-F	CACATCCAGGCTCACACCAACA	120	60
HSD11B2-R	CTCCATGCAGGTGCGGAAGTT		
GAPDH-F	GCTGATGCTCCCATGTTCGTGAT	86	60
GAPDH-R	GTGGTGCAAGAGGCATTGCTGAC		

### Lipid metabolite concentration determination

The concentrations of lipid metabolites were measured using a commercial ELISA kit (COIBO BIO, Shanghai, China) according to the manufacturer’s instructions. A standard curve was generated by plotting the known standard concentrations on the x-axis against the corresponding optical density (OD) values on the y-axis in Excel. The resulting curve equation was then used to calculate the concentration of each sample.

### Statistical analysis

All data were expressed as the mean ± SD. Statistical analysis was performed using one-way analysis of variance (ANOVA) with the SPSS 20 statistical software package (SPSS Inc., Chicago, IL, USA). The threshold of significance was defined as *p* < 0.05.

## Results

### Transcriptome and metabolome sequencing data quality

**Fig 1:**
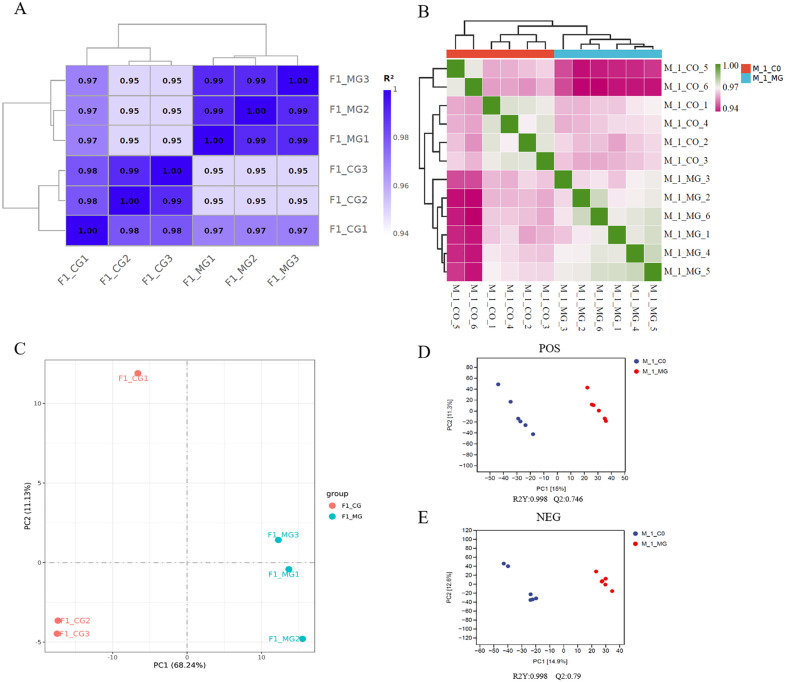
Transcriptome and metabolome sequencing data quality. **(A)** Transcriptome sample correlation analysis results. **(B)** Metabolome sample correlation analysis results. **(C)** Transcriptome sample principal component analysis results. **(D and E)** Metabolome sample partial least squares discriminant analysis results. Abbreviations: F1_CG = transcriptome sequencing co-culture goose F1 follicle GCs; F1_MG = transcriptome sequencing mono-culture goose F1 follicle GCs; M_1_CO = metabolome sequencing co-culture goose F1 follicle GCs; M_1_MG = metabolome sequencing mono-culture goose F1 follicle GCs; POS = positive ion mode; NEG = negative ion mode.

Transcriptome sequencing results showed that the samples yielded approximately 36.67 Gb of clean data. Over 95.83% of the bases in each sample achieved a quality score of Q30 or higher. The GC content across all samples was consistent, ranging from 47.97% to 49.32%. The uniquely mapped reads rate for each sample was between 81.0% and 84.14%, while the multiply mapped reads rate was below 1.64%. Sample correlation analysis revealed that all pairwise correlations among samples were greater than 0.947 (Fig 1A). Principal component analysis demonstrated a clear separation between the F1_CG (co-cultured goose F1 follicular granulosa cells for transcriptome sequencing) and F1_MG (mono-culture goose F1 follicular granulosa cells for transcriptome sequencing) groups (Fig 1C).

Metabolome sequencing results showed that the correlations among all samples were greater than 0.94 (Fig 1B). The partial least squares discriminant analysis (PLS-DA) illustrated distinct differences between M_1_CO (co-cultured goose F1 follicular granulosa cells for metabolome sequencing) and M_1_MG (mono-cultured goose F1 follicular granulosa cells for metabolome sequencing) (Fig 1D, 1E).

### Identification of differentially expressed genes and differential metabolites

**Fig 2:**
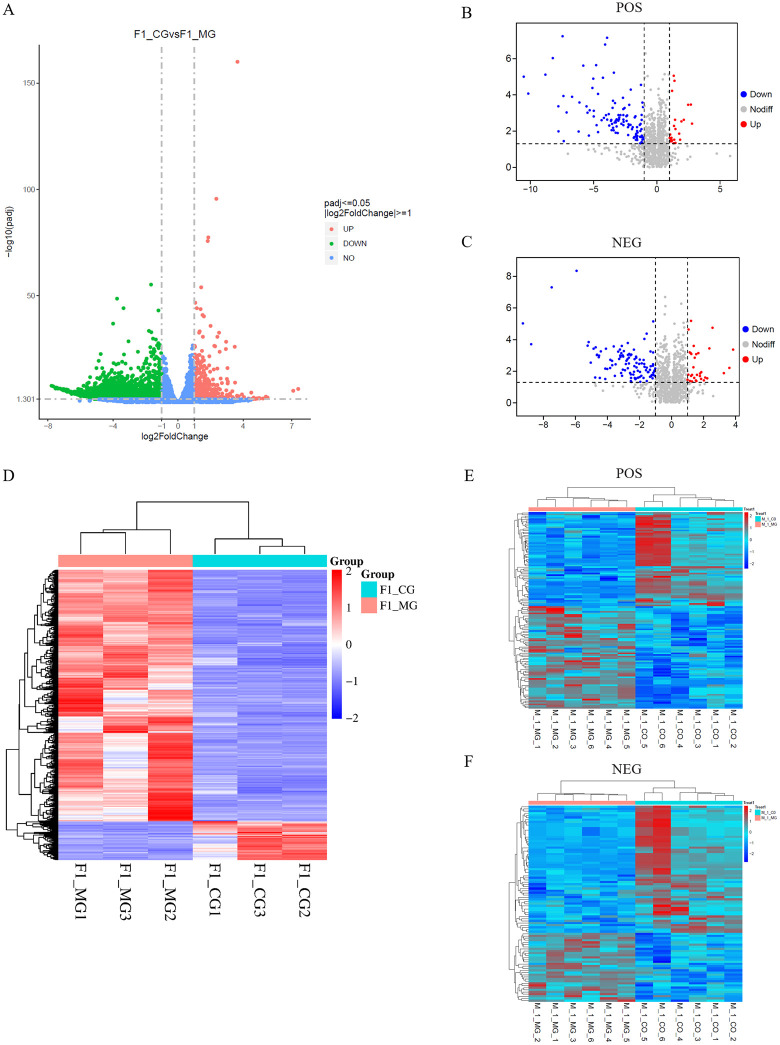
Identification of DEGs and DEMs. **(A)** DEGs volcano map, red means up-regulated and green means down-regulated. **(B and C)** DEMs volcano map, red means up-regulated and blue means down-regulated. **(D)** DEGs cluster analysis heatmap. **(E and F)** DEMs agglomerate hierarchical clustering analysis heatmap. Abbreviations: F1_CG = transcriptome sequencing co-culture goose F1 follicle GCs; F1_MG = transcriptome sequencing mono-culture goose F1 follicle GCs; M_1_CO = metabolome sequencing co-culture goose F1 follicle GCs; M_1_MG = metabolome sequencing mono-culture goose F1 follicle GCs; POS = positive ion mode; NEG = negative ion mode.

To identify DEGs (differentially expressed genes), the gene expression data was normalized using DESeq2. Differential analysis revealed that a total of 3353 DEGs (*p* < 0.05) were identified in F1_CG (co-cultured goose F1 follicular granulosa cells for transcriptome sequencing) compared with F1_MG (mono-culture goose F1 follicular granulosa cells for transcriptome sequencing), of which 2907 were up-regulated and 446 were down-regulated (Fig 2A). Cluster analysis was performed on the FPKM values of these DEGs, and the resulting heatmap displays high expression in red and low expression in blue (Fig 2D).

A total of 123 differential metabolites (DEMs) were identified in the positive ion mode (POS), consisting of 64 up-regulated and 59 down-regulated DEMs (*p* < 0.05) (Fig 2B). Additionally, 91 DEMs were identified in the negative ion mode (NEG), comprising 32 up-regulated and 59 down-regulated DEMs (*p* < 0.05) (Fig 2C). Agglomerative hierarchical clustering analysis was performed on these DEMs, and the resulting heatmaps display high abundance in red and low abundance in blue (Fig 2E, 2F).

### Enrichment analysis of DEGs and DEMs

**Fig 3:**
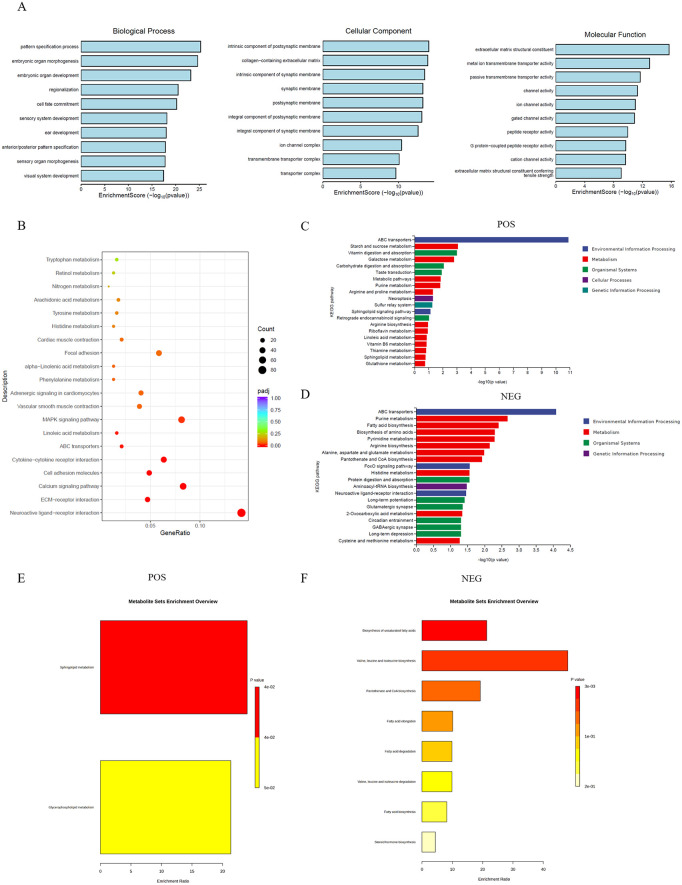
Enrichment analysis of DEGs and DEMs. **(A)** DEGs Go enrichment analysis result. **(B)** DEGs KEGG enrichment analysis result. **(C and D)** DEMs KEGG enrichment analysis result. **(E)** Significant differential lipid metabolites (POS) MetaboAnalyst 6.0 enrichment analysis result. **(F)** Significant differential lipid metabolites (NEG) MetaboAnalyst 6.0 enrichment analysis result. Abbreviations: POS = positive ion mode; NEG = negative ion mode.

The Go enrichment analysis revealed that the DEGs were significantly enriched in 30 pathways including Biological Process, Cellular Component and Molecular Function (Fig 3A).

The KEGG enrichment analysis revealed that the DEGs enriched in 20 pathways (Fig 3B). Among these, three signaling pathways were related to lipid metabolism: linoleic acid metabolism, α-linolenic acid metabolism, and arachidonic acid metabolism.

The KEGG enrichment analysis revealed that the DEMs in positive ion mode and negative ion mode enriched in 20 pathways, respectively (Fig 3C; 3D). Among these, four pathways were identified as being involved in lipid metabolism: fatty acid biosynthesis, the sphingolipid signaling pathway, linoleic acid metabolism, and sphingolipid metabolism.

Pathway enrichment analysis of the significant differential lipid metabolites was performed using the online tool MetaboAnalyst 6.0. The results revealed that in the positive ion mode, significant differential lipid metabolites were enriched in sphingolipid metabolism and glycerophospholipid metabolism pathways (Fig 3E). In the negative ion mode, the significant differential lipid metabolites were enriched in eight pathways (Fig 3F).

### Verification of sequencing results

**Fig 4:**
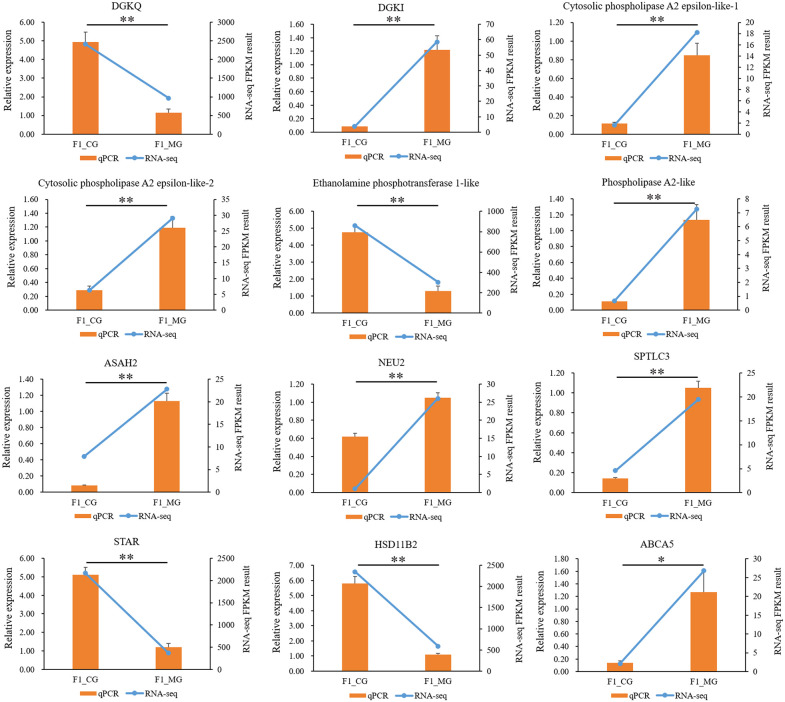
QPCR and transcriptome sequencing joint mapping. The result of qPCR and transcriptome sequencing showed that mRNA expression level of DGKQ, Ethanolamine phosphotransferase 1-like, STAR and HSD11B2 in co-culture goose F1 follicle GCs group were significantly higher than those in mono-culture goose F1 follicle GCs group (*p* < 0.05); the mRNA expression level of DGKI, Cytosolic phospholipase A2 epsilon-like-1, Cytosolic phospholipase A2 epsilon-like-2, Phospholipase A2-like, ASAH2, NEU2, SPTLC3 and ABCA5 in co-culture goose F1 follicle GCs group were significantly lower than those in mono-culture goose F1 follicle GCs group (*p* < 0.05). Abbreviations: F1_CG = transcriptome sequencing co-culture goose F1 follicle GCs; F1_MG = transcriptome sequencing mono-culture goose F1 follicle GCs. * *p* < 0.05, ** *p* < 0.01.

We detected the expression of 12 core genes from glycerophospholipid and sphingolipid metabolism pathways by qPCR. The result showed that the expression pattern of 12 genes was consistent with transcriptome sequencing. Both qPCR and transcriptome sequencing revealed that mRNA expression level of *DGKQ*, *Ethanolamine phosphotransferase 1-like*, *STAR* and *HSD11B2* in co-culture goose F1 follicle GCs group were significantly higher than those in mono-culture goose F1 follicle GCs group (*p* < 0.05). Conversely, the mRNA expression level of *DGKI*, *Cytosolic phospholipase A2 epsilon-like-1*, *Cytosolic phospholipase A2 epsilon-like-2*, *Phospholipase A2-like*, *ASAH2*, *NEU2*, *SPTLC3* and *ABCA5* in co-culture goose F1 follicle GCs group were significantly lower than those in mono-culture goose F1 follicle GCs group (*p* < 0.05) (Fig 4).

**Fig 5:**
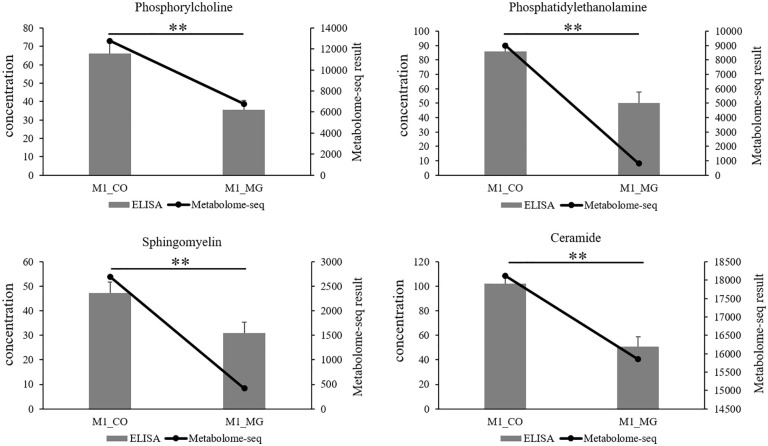
ELISA and Metabolome sequencing joint mapping. The result of ELISA and metabolome sequencing showed that the concentration of Phosphorylcholine, Phosphatidylethanolamine, Sphingomyelin and Ceramide in co-culture goose F1 follicle GCs group were significantly higher than those in mono-culture goose F1 follicle GCs group (*p* < 0.05). Abbreviations: M1_CO = metabolome sequencing co-culture goose F1 follicle GCs; M1_MG = metabolome sequencing mono-culture goose F1 follicle GCs. * *p* < 0.05, ** *p* < 0.01.

We selected 4 significantly differential lipid metabolites for concentration determination via ELISA. The result showed that the concentration of Phosphorylcholine (PC), Phosphatidylethanolamine (PE), Sphingomyelin (SM) and Ceramide in M1_CO group (co-cultured goose F1 follicle GCs for metabolome sequencing) were significantly higher than those in M1_MG (mono-cultured goose F1 follicle GCs for metabolome sequencing) group (*p* < 0.05). This result was consistent with the metabolome sequencing results (Figs 5).

### Integrative analyses of transcriptome and metabolome

**Fig 6:**
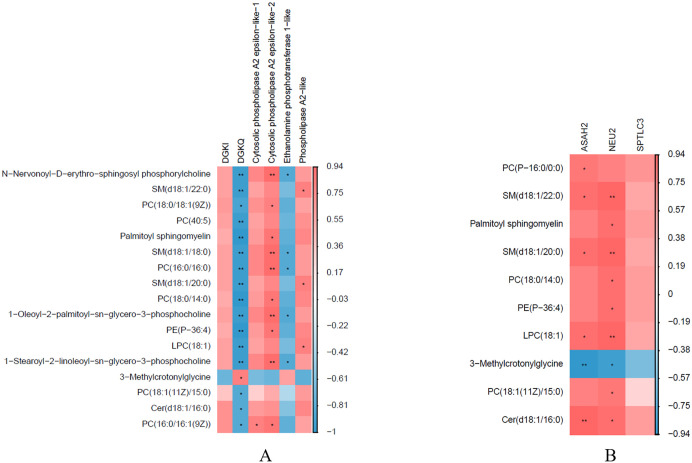
The correlation analysis of critical genes and significantly differential lipid metabolites. **(A)** Correlation analysis between phospholipid biosynthesis pathways critical genes and significantly different lipid metabolites. **(B)** Correlation analysis between sphingolipid metabolism pathways critical genes and significantly different lipid metabolites. * *p* < 0.05, ** *p* < 0.01.

Correlation analysis between critical genes in glycerophospholipid metabolism pathways and significantly different lipid metabolites revealed that *Cytosolic phospholipase A2 epsilon-like-1*, *Cytosolic phospholipase A2 epsilon-like-2* and *Phospholipase A2-like* significantly and positively correlated with significantly differential lipid metabolites. In contrast, *DGKQ* and *Ethanolamine phosphotransferase 1-like* were significantly and negatively correlated with significantly differential lipid metabolites except 3-Methylcrotonylglycine (*p* < 0.05) (Fig 6A). In sphingolipid metabolism pathways *ASAH2* and *NEU2* were significantly positively correlated with the significantly differential lipid metabolites except 3-Methylcrotonylglycine (*p* < 0.05) (Fig 6B).

## Discussion

As a key component of the follicle, GCs play an important role in its growth and development [26–28]. TCs and GCs are located on opposite sides of the follicular basal lamina, and they can communicate with each other to influence follicular development [14–16,29,30]. Lipid metabolism in GCs is critical for follicle development, a process that also depends on the role of TCs. However, the underlying mechanisms have not been fully elucidated.

In the present study, Transcriptome and metabolome analyses were performed on co-cultured and mono-cultured goose F1 follicle theca cells (TCs) and granulosa cells (GCs). Sample correlation and principal component analyses of the resulting data showed that the correlations among all samples were greater than 0.94 (Fig 1A, 1B). Principal component analysis also revealed clear separation between the co-culture and mono-culture groups (Fig 1C, 1D, 1E). The transcriptome analysis identified 3,353 differentially expressed genes (DEGs) (Fig 2A), while the metabolome analysis identified 123 differentially expressed metabolites (DEMs) in positive ion mode (Fig 2B) and 91 DEMs in negative ion mode (Figs 2C). These results indicate that TCs significantly influence both the gene expression and metabolite profiles of GCs, further confirming the important role of TCs in regulating GCs.

The KEGG enrichment analysis revealed that the DEGs were significantly enriched in 3 lipid metabolism signaling pathways: Linoleic acid metabolism, alpha−Linolenic acid metabolism and Arachidonic acid metabolism signaling pathway (Fig 3B). We then screened significant differential lipid metabolites for enrichment analysis in positive and negative ion modes using the online tool MetaboAnalyst 6.0. The results showed that a large number of differential lipid metabolites were enriched in Sphingolipid metabolism and Glycerophospholipid metabolism pathways in GCs co-culture group at positive ion mode (Table 2) (Fig 3E). In the negative ion mode the significant differential lipid metabolites were enriched in 8 pathways (Fig 3F). The combined enrichment results from the transcriptome and metabolome analyses suggest that the effect of TCs on GCs is closely related to lipid metabolism.

**Table 2:** KEGG enrichment signaling pathways.

Ion mode	Signaling pathways name	KEGG enrichment P value	Related differential lipid metabolites
positive ion mode	Sphingolipid metabolism	0.0412	SM(d18:1/22:0); SM(d18:1/18:0); SM(d18:1/20:0)
	Glycerophospholipid metabolism	0.0463	PC(P-16:0/0:0); PC(18:0/18:1(9Z)); PC(40:5); PC(16:0/16:0); PC(18:0/14:0); LPC(18:1); PC(18:1(11Z)/15:0);PC(18:4(6Z,9Z,12Z,15Z)/P-16:0); PC(16:0/16:1(9Z))
negative ion mode	Biosynthesis of unsaturated fatty acids	0.0031	Palmitic acid; Stearic acid
	Valine, leucine and isoleucine biosynthesis	0.0207	alpha-Ketoisovaleric acid

Moreover, the enrichment analysis of the differential lipid metabolites showed that a large number of differential lipid metabolites were enriched in sphingolipid metabolism and glycerophospholipid metabolism pathways (Table 2). Therefore, we further verified and analyzed the core lipid metabolites and genes in these two metabolic pathways using ELISA and qPCR. Four metabolites from the glycerophospholipid metabolism and sphingolipid metabolism pathways significantly differential lipid metabolites were selected for concentration determination by ELISA. The result showed that the concentration changes of Phosphorylcholine (PC), Phosphatidylethanolamine (PE), Sphingomyelin (SM) and Ceramide were consistent with the metabolome analysis results, as they were all significantly upregulated in the co-culture group (Fig 5). It is well-established that phospholipids are important components of all cellular membranous organelles and are extensively involved in nutrient transport and signal exchange [12,31–34]. These results suggest that the effect of TCs on GCs lipid metabolism may through promote GCs glycerophospholipid metabolism and sphingolipid metabolism.

**Fig 7:**
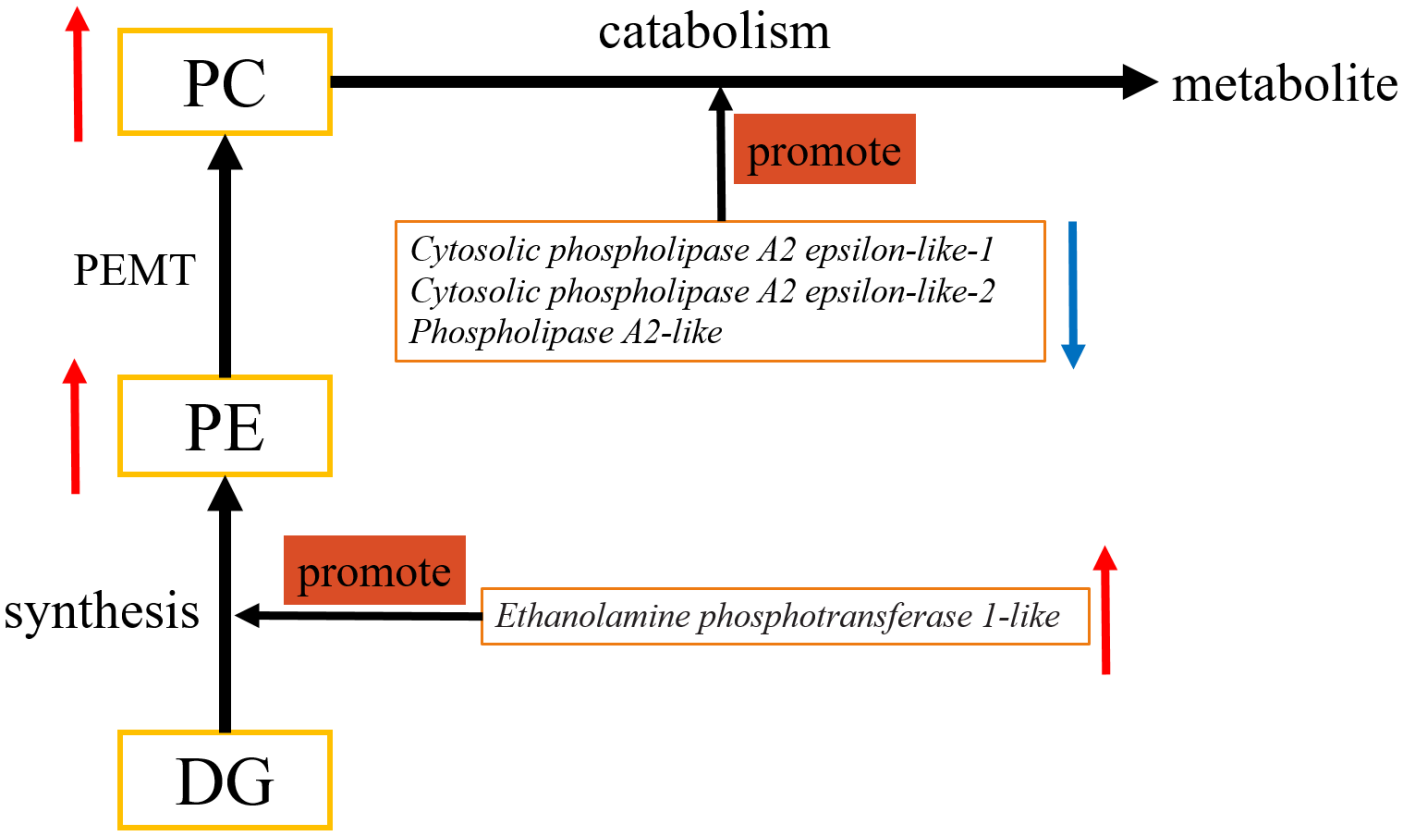
Changes of metabolite concentration and mRNA expression levels of related core genes at glycerophospholipid metabolism pathway in goose F1 follicle GCs co-culture group. Abbreviations: DG, diacylglycerol; PC, phosphatidylcholine; PE, phosphatidylethanolamine; PEMT, phosphatidylethanolamine N-methyltransferase; SM, sphingomyelin.

Previous research indicates that PC is the most abundant phospholipid in cells, accounting for 40%–60% of total phospholipids, and is catabolized by phospholipase A2 [31,35]. In goose F1 follicle GCs co-culture group the concentration of PC significantly higher than in mono-culture group. Therefore, we further examined the genes closely related to PC catabolism (Fig 6A). The result shows that the mRNA expression level of *Cytosolic phospholipase A2 epsilon-like-1*, *Cytosolic phospholipase A2 epsilon-like-2* and *Phospholipase A2-like* gene which encoding Phospholipase A2 in goose F1 follicle GCs co-culture group were significantly lower than in mono-culture group. This finding is consistent with the mechanism by which PC is catabolized by phospholipase A2 (Fig 7) [35]. Moreover, PE is the second most abundant phospholipid in mammalian cell membranes and constitutes about approximately 45% of the total phospholipids [32]. The synthesis of PE requires Ethanolamine phosphotransferase 1 [32,36]. In goose F1 follicle GCs co-culture group the concentration of PE significantly higher than that in mono-culture group. Therefore, we further examined the genes closely related to PE synthesis (Fig 6A), the result shows that the mRNA expression level of *Ethanolamine phosphotransferase 1-like* gene which encoding Ethanolamine phosphotransferase 1 in goose F1 follicle GCs co-culture group significantly higher than mono-culture group. This result consisted with synthesized of PE requires Ethanolamine phosphotransferase 1 (Fig 7) [36]. Additionally, PE can be converted to PC [31]. We measured the PEMT gene expression by qPCR (S1 Fig). The mRNA expression level of PEMT gene in co-culture goose F1 follicle granulosa cells (GCs) group was significantly higher than that from the mono-culture group (*p* < 0.05). This finding is consistent with the known mechanism in mammals, where phosphatidylcholine (PC) is also synthesized via phosphatidylethanolamine N-methyltransferase (PEMT) [31,35]. Therefore, combined with the physiological process of lipid metabolism and transcriptome/qPCR and metabolome/ELISA results, we drew a model map of TCs regulation of GCs lipid metabolism related pathways (Fig 7). We propose that in goose F1 follicles, TCs influence GC glycerophospholipid metabolism by promoting PE synthesis and inhibiting PC catabolism.

## Conclusion

In this study, we investigated the effect of TCs on GCs lipid metabolism based on in vitro co-culture model. Our results revealed that TCs could significantly change the metabolite and gene expression profiles of GCs, and the results of transcriptome/qPCR and metabolome/ELISA all showed that TCs promoting PE synthesis and inhibit PC catabolism of GCs. In conclusion, these findings suggest that TCs modulate the lipid metabolism of GCs in goose F1 follicles through the promotion of PE synthesis and inhibition of PC catabolism within the glycerophospholipid metabolism pathway.

## Supporting information

S1 Fig The qPCR result of *PEMT* gene expression. Abbreviations: F1_CG = co-culture goose F1 follicle GCs; F1_MG = mono-culture goose F1 follicle GCs. * *p* < 0.05, ** *p* < 0.01. (DOCX)

## Methods statement

We declare that all methods were carried out in accordance with relevant guidelines and regulations and all methods are reported in accordance with submission guidelines.
