# Supplementary material for: Comprehensive transcriptome and metabolome analysis to exploration the effects of TCs on GCs lipid metabolism at goose pre-ovulatory follicle
Source: PLoS One. 2026 Jan 9;21(1):e0340283. doi: 10.1371/journal.pone.0340283 (PMC12788666; doi:10.1371/journal.pone.0340283)
Supplement: S1 Fig — (DOCX) [file pone.0340283.s001.docx]

Supporting Information for Comprehensive transcriptome and metabolome analysis to exploration the effects of TCs on GCs lipid metabolism at goose pre-ovulatory follicle


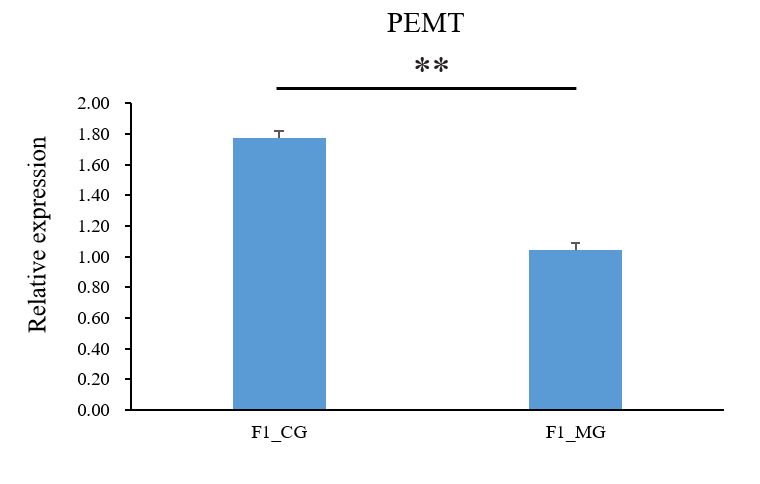


Figure S1. The qPCR result of *PEMT* gene expression.

Abbreviations: F1_CG = co-culture goose F1 follicle GCs; F1_MG = mono-culture goose F1 follicle GCs. * *p* < 0.05, ** *p* < 0.01.
